# Scurvy, Starvation, and Flea Infestation – A Case Report From 21st Century Europe

**DOI:** 10.7759/cureus.13158

**Published:** 2021-02-05

**Authors:** Alexandra Esteves, Francisco Teixeira da Silva, José Carvalho, Paula Felgueiras, Paulo Laranjeira

**Affiliations:** 1 Internal Medicine, Unidade Local de Saúde do Alto Minho, Viana do Castelo, PRT; 2 Department of Safety, Health and Environment, School of Management and Technology, Polytechnic Institute of Porto, Porto, PRT

**Keywords:** flea infestations, malnutrition, avitaminosis, scurvy, social determinants of health

## Abstract

Scurvy is a disease caused by vitamin C deficiency, historically associated with long sea voyages, periods of famine and war. Currently, it is often misdiagnosed and underreported, as physicians tend to consider it a disease of the past.

We present the case of a 79-year-old female who was admitted to the Emergency Department complaining of pruritus. The patient lived alone and in poor hygiene conditions. Diet was scarce. One week before admission she was in contact with flea-ridden stray dogs. Sometime later she noted several fleas and multiple pruritic small papules, crusts, and excoriations on her torso, limbs, palms, soles, and scalp. Physical examination showed the patient to be pale, emaciated, and poorly groomed. Laboratory analysis showed microcytic anemia. Flea bites were treated with oral antihistamines and prednisolone. Follow-up one month later showed weight gain and partial resolution of skin lesions. However, multiple small ecchymosis on both lower and upper limbs, and occasional perifollicular petechias on her lower extremities, were noted. Additional workup showed undetectable serum vitamin C levels. A diagnosis of scurvy was made. The patient was treated with 1000 mg per day of oral vitamin C for one month leading to complete recovery.

We present this case to remind that early recognition of vitamin C deficiency and appropriate supplementation are essential in patients with scurvy. Physicians should be aware of classic signs, symptoms, and social factors associated with this forgotten disease.

## Introduction

Individual health conditions and outcomes are shaped by biological, as well as social, environmental, cultural, political, and economic factors. These include access not only to quality housing, healthcare, education, work conditions, but also to environmental quality, sanitation availability, safety, and food [1].

Scurvy is a disease caused by vitamin C deficiency, historically associated with long sea voyages, periods of famine and war [2]. During the 20th century vitamin C deficiency has significantly decreased. Nevertheless, population studies in industrialized societies in North America and Europe still report prevalence ranging from 3% to 25.3% [3-6].

At-risk populations include low-income patients (poor access to groceries), adults living alone (most commonly male, bachelors, or widowers), patients with psychiatric or behavioural disorders, cancer, inflammatory bowel disease, peptic disease, or gastroesophageal reflux. Poor or absent dentition, bizarre dietary beliefs, nutritional ignorance, diet fads, and taste preferences further challenge regular consumption of fruit and vegetables. The most common behavioural disorder associated with scurvy is alcoholism [7,8].

Scurvy is frequently misdiagnosed and underreported, misleading physicians to consider it a disease of the past. We present the case of a malnourished 79-year-old patient with scurvy, and a summary of the pathophysiology and therapeutic options.

## Case presentation

A 79-year-old female was admitted to a Portuguese Emergency Department (ED) due to complaints of pruritus. She had a previous history of arterial hypertension and alcoholism, though she was abstinent for more than 40 years. Regular medication was amlodipine 5mg once a day. No allergies, tobacco, toxins, drug exposure, or other medical history were known. The patient lived alone in a suburban apartment, with extremely poor conditions of insulation and hygiene. She had contact with stray dogs and cats. Income was insufficient and irregular. 

As the patient was edentulous, her diet consisted of liquid dairy products and sugary processed foods, and was irregular due to frequent income shortage.

One week before admission she had contact with presumably flea-infected stray dogs. A few hours later she noted multiple pruritic small papules on her torso, limbs, palms, soles, and scalp. Distribution did not follow any specific pattern. Multiple crusts and excoriations soon developed due to scratching. As the pruritus became unbearable, she was admitted to the ED.

On admission, she complained of generalized pruritus and hunger. Detailed medical history was negative for any other symptoms, namely fever, asthenia, dyspnoea, bleeding, melena, dysphagia, nausea, vomit, diarrhoea, obstipation, arthralgia, thoracic or abdominal pain, on admission or on the previous days. On physical examination the patient was pale, emaciated, and poorly groomed. Reported weight was 32 Kg for a height of 158 cm (body mass index (BMI) of 12.8). Besides the small erythematous papules, crusts, and excoriations previously described, several fleas were identified. She otherwise appeared comfortable. Blood pressure (BP) and heart rate (HR) were stable (BP: 143/67 mmHg and HR: 92 bpm), peripheral oxygenation saturation 100% and she was afebrile. No other abnormalities were noted.

Laboratory analysis revealed microcytic anemia (hemoglobin: 8.2 g/dL, median corpuscular volume: 66 fL) and leucocytosis (white blood cells: 12.81 x 10^9^/L). Serum iron concentration was low (iron: 10 µ/dL) and total iron-binding capacity was elevated (total iron-binding capacity: 377 µ/dL). Serum ferritin, folic acid, vitamin B12, bilirubin, aspartate aminotransferase, alanine aminotransferase, alkaline phosphatase, and gamma-glutamyl transferase were normal and no other abnormalities were detected. In the ED she was showered, new clothes were given, and she was fed. Oral antihistamines and 20mg of oral prednisolone were prescribed with marked improvement from the pruritus. Hospital discharge was possible, on next day, after ensuring safe housing conditions with a sensible family member. Oral iron supplements were prescribed, and a complete diet rich in iron was recommended.

**Figure 1:**
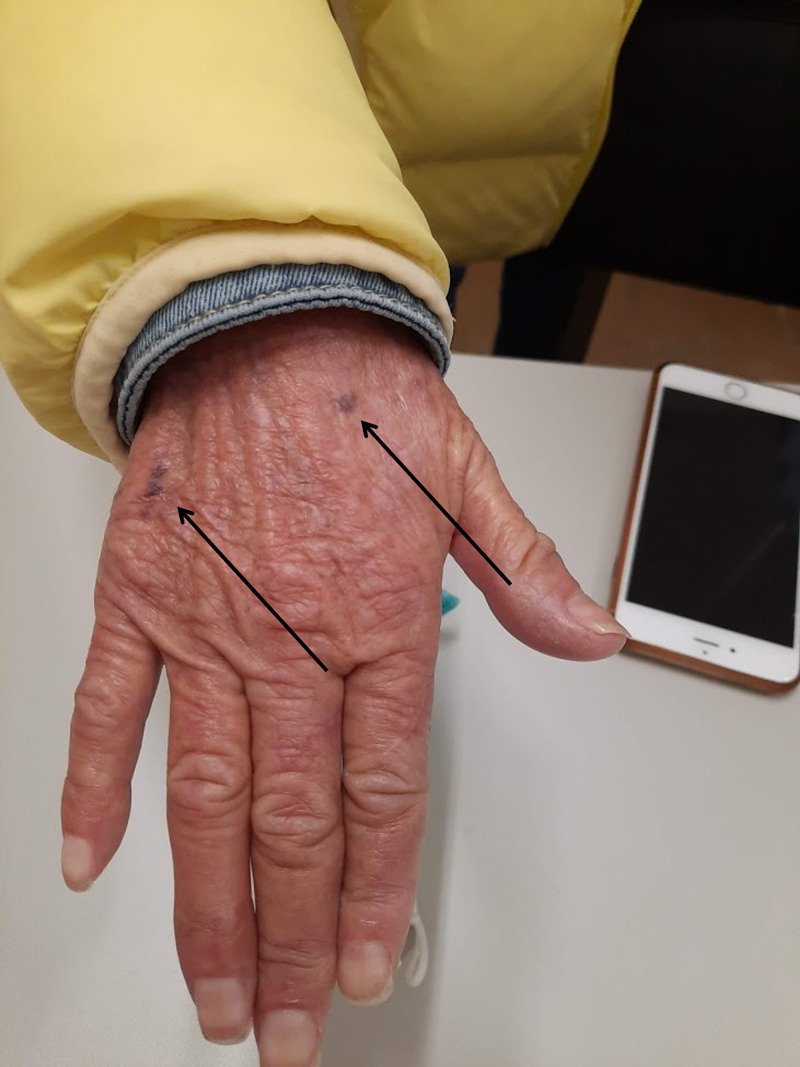
Small ecchymoses on the right hand (arrows)

**Figure 2:**
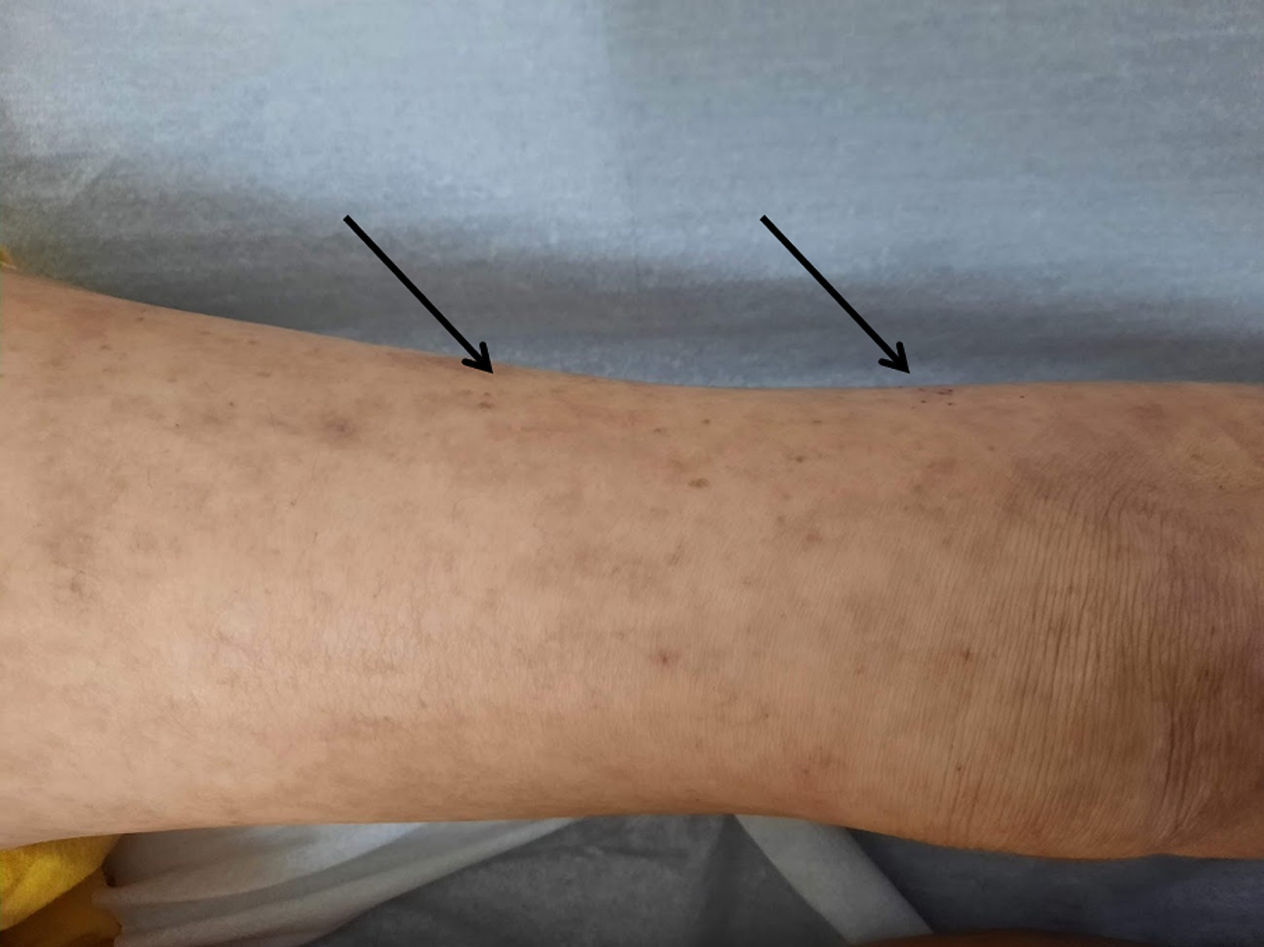
Scarce perifollicular petechias on lower limb (arrows)

**Figure 3:**
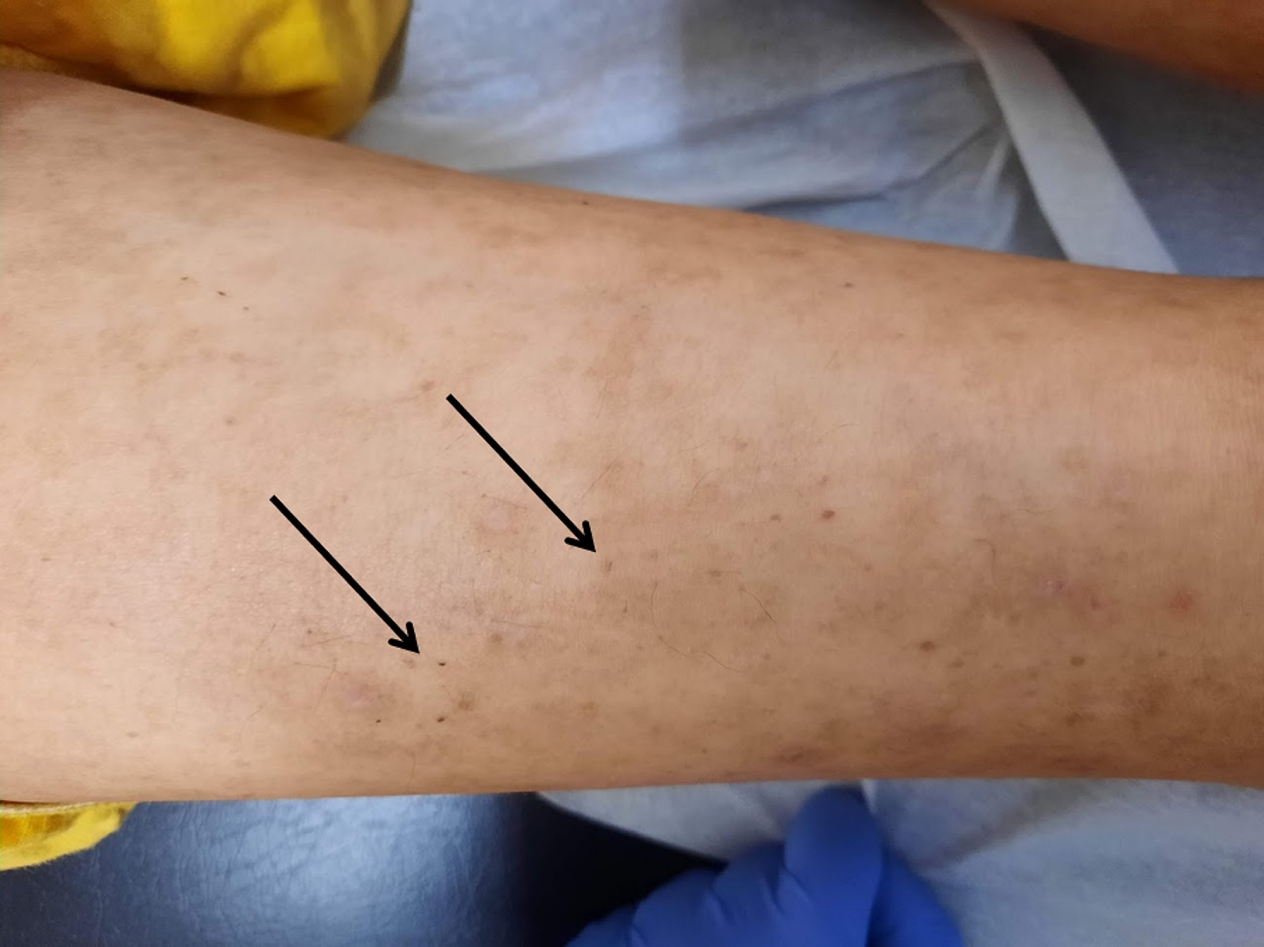
Curly corkscrew body hairs (arrows) on lower limb

One month later on follow-up she showed partial recovery. The patient was living with a niece who revealed difficulty to improve dietary routines because of patient's refusal to eat certain foods (mainly fruits and vegetables). She had gained 1 Kg (BMI: 13.2) and most of the papules, crusts, and excoriations had disappeared. Nevertheless, multiple small ecchymosis on both lower and upper limbs (Figure 1), scarce perifollicular petechias on her lower extremities (Figure 2), and sporadic curly corkscrew body hairs were noted (Figure 3). Besides poor oral hygiene and being edentulous, oral examination did not reveal any relevant findings. Her lung, heart, abdominal, and neurologic examinations were unremarkable. Additional workup (Table 1) was ordered and returned 10 days later which showed undetectable serum vitamin C levels. A diagnosis of scurvy was made. No other vitamin deficiencies (vitamin A, B1, B3, B6, and D) were reported.

**Table 1:** Laboratory data ALP: alkaline phosphatase; ALT: alanine aminotransferase; AST: aspartate aminotransferase; GGT: gamma-glutamyl transferase; LDH: lactate dehydrogenase, MCV: mean corpuscular volume; TIBC: total iron binding capacity, HDL: high-density lipoprotein, LDL: low-density lipoprotein

Variable	Reference range (adult)	Results
Hemoglobin (g/dL)	11.8 - 15.8	8.2
Hematocrit (%)	36.0 - 46.0	30.3
Red blood cells (10^6/µL)	4.2 - 5.4	4.55
MCV (fL)	80.4 - 96.4	66.6
White cell count (10^3/µL)	4.0 - 10.0	12.82
Differential cell count (10^3/L)		
Neutrophils	1.5 - 8.0	10.9
Lymphocytes	0.8 - 4.0	0.8
Platelet count (10^3/µL)	150 - 400	409
Reticulocytes (%)	0.5 - 1.5	0.91
Peripheral blood smear	Moderate anysocitosis. Moderate erythrocyte dysmorphism. Moderate anisocrhomia. Moderate hypochromia.	
Glucose (mg/dL)	70 - 110	170
Glycated hemoglobin (%)	<65%	4.70%
Urea (mg/dL)	17.0 - 43.0	48
Creatinine (mg/dL)	0.6 - 1.0	1.02
Sodium (mmol/L)	136 - 145	139
Potassium (mmol/L)	3.5 - 5.1	3.9
Calcium (mg/dL)	8.6 - 10.3	9.9
Bilirubin (mg/dL)		
Total	0.3 - 1.2	0.4
Direct	<0.5	0.14
LDH (UI/L)	125 - 220	153
ALP (UI/L)	30 - 120	110
GGT (UI/L)	<38	9
ALT (UI/L)	7 - 45	14
AST (UI/L)	8 - 35	7
Iron (µ/dL)	70 - 180	10
Ferritin (ng/mL)	4.6 - 204	48.6
TIBC (µ/dL)	250 - 245	377
Folic acid (ng/mL)	2.34 - 17.56	3.4
Vitamins		
Vitamin A (mg/L)	0.3 - 1	0.76
Vitamin B1 (µg/dL)	2 - 7.2	5.6
Vitamin B3 (µg/mL)	8 - 52	16
Vitamin B6 (nmol/L)	23 - 172.5	28.7
Vitamin B12 (pg/mL)	187 - 883	383
Vitamin C (mg/dL)	0.4 - 2	<0.1
Vitamin D (ng/mL)	6.2 - 45.5	25.3
Total cholesterol (mg/dL)	< 200	226
HDL (mg/dL)	> 60	56
LDL (mg/dL)	< 100	147
Protein electrophoresis	No monoclonal spikes	
Total proteins (g/dL)	6.4 - 8.2	7.6
Albumin (g/dL)	3.5 - 5.2	4.4

The patient was treated with vitamin C, at a dose of 1000mg per day for a month. A diet rich in fruits and vegetables was recommended, and nutritional, psychiatric, and psychological support was offered. Follow-up one month later showed both complete resolution of all her skin lesions and weight increase to 34.5 Kg (BMI: 13.8).

## Discussion

Vitamin C is a simple water-soluble sugar-like molecule present in most fruits and vegetables. Most animals can derive it from glucose via various enzyme systems. Humans, other primates, and guinea pigs are some of the few animal species that are unable to do so, and, therefore, must absorb it through dietary intake. The recommended daily allowance (RDA) of vitamin C ranges from 75 mg per day for women to 90 mg per day for men, though during lactation and other inflammatory states the RDA is increased up to 100 mg per day. A single orange contains approximately 50 mg of vitamin C and as little as 6 to 10 mg of vitamin C per day is enough to prevent clinical scurvy [9].

Vitamin C acts as an essential cofactor for the two enzymes required for collagen synthesis. It also acts as an antioxidant, reducing reactive free radical and oxygen species in oxidative states. Other actions include promotion of iron absorption, biosynthesis of carnitine, hormones (corticosteroids and aldosterone), and neurotransmitters (conversion of dopamine to norepinephrine), and metabolism of cholesterol and tyrosine. The most frequent clinical manifestations are consequences of impaired collagen synthesis, which leads to tissue and capillary fragility, tissue haemorrhage, delayed wound healing, scar dehiscence, dystrophic hair lesions, and bone fragility. Early manifestations usually appear in areas subjected to higher hydrostatic pressure, such as buttocks and lower limbs, but can be present in almost any organ [10].

Skin and integumentary system manifestations include perifollicular haemorrhage, petechiae, ecchymoses, palpable purpure, and oedema. Perifollicular haemorrhage, with corkscrew hair deformity due to follicular hyperkeratosis is virtually pathognomonic of scurvy. Oral complications are frequent and include gingival hypertrophy, chronic inflammation, intramucosal vascular fragility leading to oedema and microhaemorrhage. This, combined with poor oral hygiene and alveolar bone absorption, leads to tooth decay and loss. Rheumatological manifestations include arthralgias, usually due to hemarthrosis and subperiosteal haemorrhages. Myalgias are common and thought to be due to the reduced production of carnitine. Ocular complications include conjunctival haemorrhages, fundus changes of flame-shaped haemorrhages and cotton-wool spots. Papilledema and optic nerve atrophy can develop if bleeding locates in the retrobulbar space and optic nerve sheaths. Other associated symptoms are asthenia, depression, anorexia, sicca symptoms and peripheral neuropathy [8].

Constitutional symptoms may be secondary to anemia, which accompanies scurvy in up to 75% of patients. Anemia in vitamin C deficiency is multifactorial. Hemorrhage may lead to normochromic-normocytic anemia with elevated reticulocyte count and erythrocyte hyperplasia in bone marrow samples. Decreased inhibition of folate excretion by vitamin C and concomitant decreased folate ingestion (as vegetables and fruits rich in vitamin C are also the main sources of dietary folate) and some patients can also present with to macrocytic anemia. Hemolysis is usual, though the exact mechanism is unknown. Iron deficiency due to decreased iron absorption leads to microcytic anemia in other patients. Chest pain, hypotension, dyspnoea, cyanosis, ST-segment elevation, and first-degree heart block have all been described, and have been also associated to heart failure, pulmonary hypertension, hemopericardium, impaired catecholamine synthesis and weakened vasomotor response [7,11,12].

The differential diagnosis of scurvy is broad and includes haematological abnormalities, medication side effects, infections, ulcerative gingivitis, vascular disorders, other vitamin deficiencies and trauma. Common misdiagnoses include vasculitis, blood dyscrasias, and ulcerative gingivitis, making high suspicion and clinical recognition central to avoid unnecessary complementary exams and inappropriate treatments [10].

Diagnosis of scurvy is clinical, based on dietary history, clinical features, and resolution of signs after vitamin C supplementation. Serum levels of vitamin C below 0.2 mg/dL suggest scurvy and are useful to confirm atypical cases. At these concentrations, fatigue is invariably present, and other symptoms and signs will appear soon after [9]. Nevertheless, normal serum values cannot exclude this diagnosis, as they tend to only reflect recent dietary intake. Leucocyte vitamin C levels reflect more accurately body stores, but its measurement is difficult to perform and not readily available [8].

Treatment is based on appropriate dietary adjustments and vitamin C supplementation. Consumption of five servings of fruit and vegetables a day will not only exceed the RDA for vitamin C, but it also has the benefit of treating other common concomitant vitamin deficiencies that may be present. Dose and duration of vitamin C supplementation should be individualized, and recommendations vary [10]. Oral vitamin C is well absorbed at lower doses, but absorption decreases in a sigmoidal relationship as the dose increases. Doses greater than 500 mg per day contribute little to plasma or tissue stores, and complete plasma saturation occurs at 1000 mg per day [13]. Adults are usually treated with 500 to 1000 mg per day of oral vitamin C for a one month or until full recovery occurs [14]. Other schemes include 1000 mg per day for the first three to five days followed by 300 to 500 mg per day for at least a week [10], or 300 mg three times daily for three months [8].

Acute treatment of scurvy supplementation carries an excellent prognosis even in severe disease. Except for lost teeth, no permanent sequalae are known. Symptoms usually resolve in one to five days, most physical findings in one to two weeks, normal hair growth resumes in four weeks, and complete recovery usually occurs before three months of treatment [8,10].

In our patient, a concomitant flea infestation masqueraded scurvy manifestations. Fleabites are characterized by sudden-onset multiple pruritic papules with haemorrhagic crusts appearing in exposed areas of skin. Some patients present severe hypersensitivity reactions resembling urticaria, and persistent infestations with fleas can lead to papular urticaria and psychological distress. Symptomatic fleabites are usually treated with oral antihistamines and topical corticosteroids. As important vectors for infectious diseases (*Bartonella henselae*, *Rickettsia typhi*, *Rickettsia felis*, and *Yersinia pestis*), treatment must include eradication of fleas both in the patient and in domestic reservoirs [15].

## Conclusions

Clinical manifestations of scurvy are time-dependent and proportional to the body's vitamin C deficit. Both mild cases, presenting subtle and nonspecific symptoms such as fatigue and myalgia, as well as severe cases, presenting haemorrhagic and cardiovascular complications, are often misdiagnosed. Direct sequalae from scurvy after appropriate treatment are mainly related to tooth loss, as treatment is extremely safe and effective even in advanced disease. Therefore, severe adverse outcomes are usually associated with complications related to delayed diagnosis, and to unnecessary or inappropriate tests, treatments, or procedures. Classic signs and symptoms should be straightforwardly recognized by physicians and vitamin C supplementation immediately started in suspicious cases. Laboratory confirmation is not essential for diagnosis, though it is useful in patients with atypical or mild signs. Though early recognition of vitamin C deficiency and supplementation is essential, scurvy arises from the sum of biological and social factors, hence addressing both is fundamental for a favourable outcome.
